# Age-Related Changes in the Daily Rhythm of Photoreceptor Functioning and Circuitry in a Melatonin-Proficient Mouse Strain

**DOI:** 10.1371/journal.pone.0037799

**Published:** 2012-05-22

**Authors:** Kenkichi Baba, Francesca Mazzoni, Sharon Owino, Susana Contreras-Alcantara, Enrica Strettoi, Gianluca Tosini

**Affiliations:** 1 Circadian Rhythms and Sleep Disorders Program, Neuroscience Institute, Department of Anatomy and Neurobiology, Morehouse School of Medicine, CNR, Pisa, Italy; 2 Neuroscience Institute, Italian National Research Council, CNR, Pisa, Italy; Institut National de la Recherche Agronomique-CNRS UMR6175, France

## Abstract

Retinal melatonin is involved in the modulation of many important retinal functions. Our previous studies have shown that the viability of photoreceptors and ganglion cells is reduced during aging in mice that lack melatonin receptor type 1. This demonstrates that melatonin signaling is important for the survival of retinal neurons. In the present study, we investigate the effects of aging on photoreceptor physiology and retinal organization in CH3-f+/+ mice, a melatonin proficient mouse strain. Our data indicate that the amplitude of the a and b waves of the scotopic and photopic electroretinogram decreases with age. Moreover, the daily rhythm in the amplitude of the a- and b- waves is lost during the aging process. Similarly, the scotopic threshold response is significantly affected by aging, but only when it is measured during the night. Interestingly, the changes observed in the ERGs are not paralleled by relevant changes in retinal morphological features, and administration of exogenous melatonin does not affect the ERGs in C3H-f^+/+^ at 12 months of age. This suggests that the responsiveness of the photoreceptors to exogenous melatonin is reduced during aging.

## Introduction

In the mammalian retina, melatonin is synthesized by photoreceptors with high levels of melatonin at night and lower levels during the day [1]. Melatonin in the eye is believed to be involved in the modulation of many important retinal functions; for instance, it can modulate the electroretinogram response (ERG, [2]–[5]), and administration of exogenous melatonin increases light-induced photoreceptor degeneration [6]. Melatonin may also have protective effects on photoreceptors [4], [7] and on other cell types, such as ganglion cells [4]. Recent studies have implicated melatonin in the pathogenesis of age-related macular degeneration (AMD). Yi et al. [8] reported that oral administration of melatonin (3 mg) may protect the retina and delay the progression of AMD, while Rosen et al. [9] reported that production of melatonin is decreased in AMD patients with respect to age-matched controls, thus suggesting that a deficiency in melatonin may play a role in the pathogenesis of AMD.

Melatonin acts via melatonin receptors that are found in many retinal cells types [10]. In particular, melatonin receptors type 1(MT_1_) have been localized to the photoreceptor cells in many species, including humans [4], [11], [12]; thus, this neurohormone may play an important role in photoreceptor functions.

Previous studies have investigated the effect of aging on the retinal structure and functioning in the mouse. Overall, the data indicate that the amplitude of the ERG declines with age and that these changes do not correlate with significant changes in the morphology of the photoreceptor cells, at least until 12 months of age (13, 14). However, it is important to note that these studies were performed in C57BL/6 and Balb/c mice, which are genetically deficient to synthesize melatonin in the pineal gland and retina (15, 16) because they have a mutation in AANAT that prevents the synthesis of appreciable amounts of melatonin (17).

Several studies have indicated that melatonin may delay the neurodegenerative process of aging [18]. Therefore, we investigate the effects of aging on retinal functioning and organization in C3H-f+/+ mice, a melatonin proficient mouse strain [15], [16].

## Results

### Effect of Aging on the Scotopic Electroretinogram (ERG) and Scotopic Threshold Response (STR)

**Figure 1:**
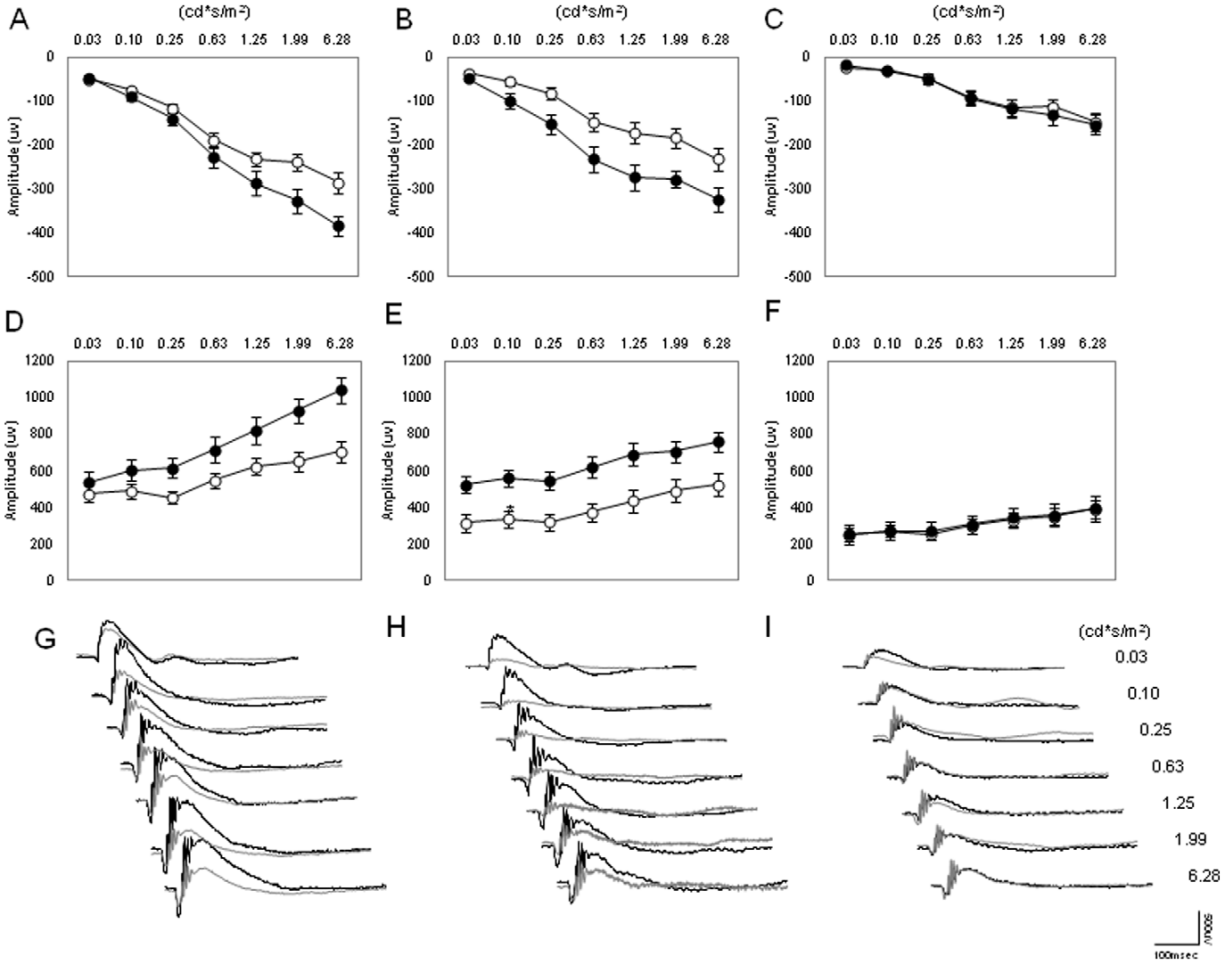
Effect of aging on the scotopic ERG and its daily rhythm. The amplitude of the a wave (A–C) and b wave (D–F) of the scotopic ERG steadily decreased during aging, and the daily rhythm in the a and b wave amplitude was only present in mice at 3 and 6 months (Two-way ANOVA, P<0.001, followed by Tukey tests). No significant difference in the amplitude of the a and b waves was detected in 12-month-old mice (Two-way ANOVA, P>0.1) White circles indicate the average amplitude at ZT6, and black circles indicate the average values at ZT18. Each point represents the mean ± SEM, where N = 4–14 for each point. Figures G through I, show representative traces of the dark-adapted ERGs at ZT18 (black lines) and at ZT6 (grey lines) for the different ages (G = 3 months; H = 6 months; I = 12 months).

**Figure 2:**
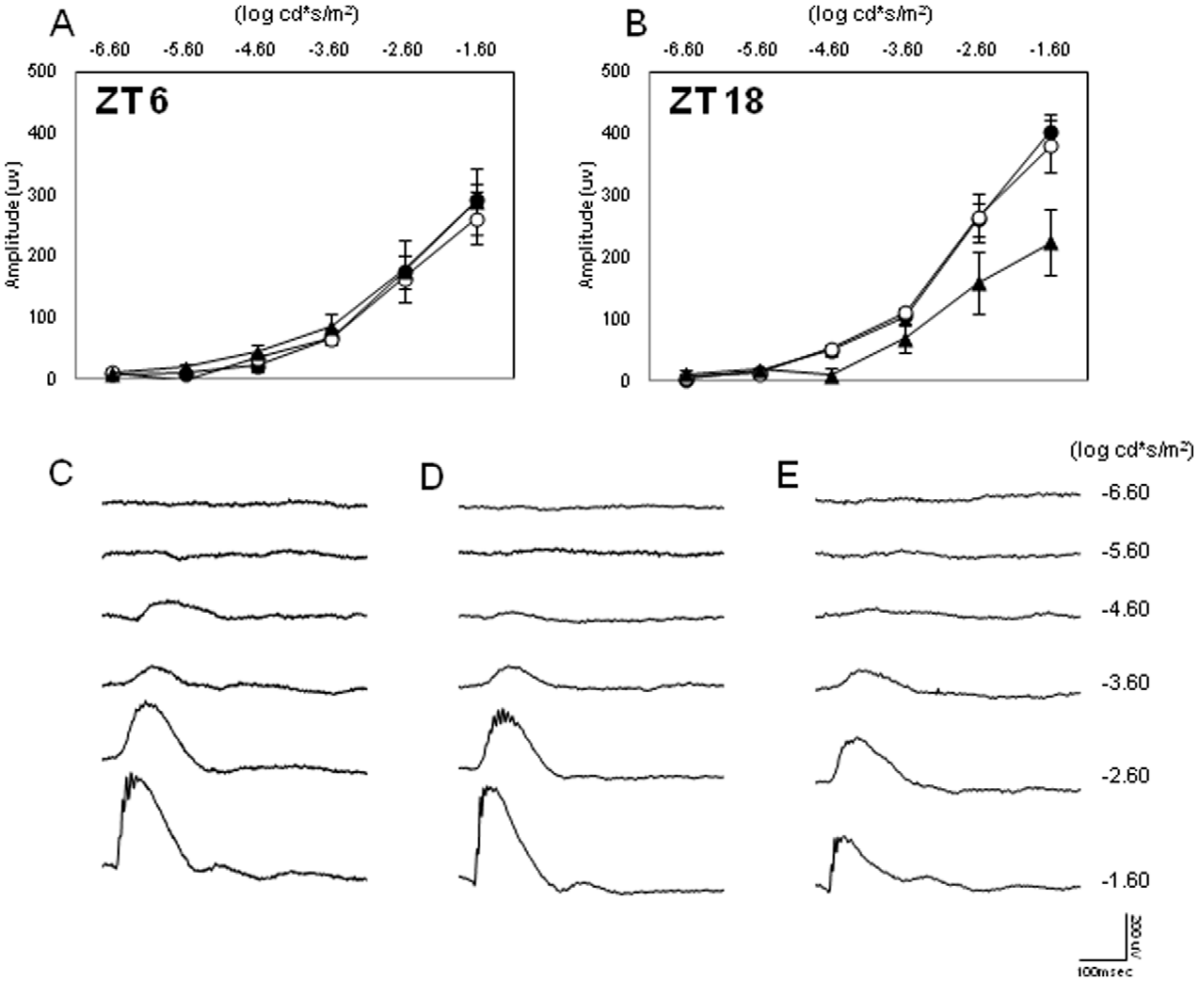
Effect of aging on the STR. (A) No differences were observed at ZT6 in the STR among young and old mice (t-tests, P>0.5 in all cases). Black circles, white circles, and black triangles indicate 3-, 6-, and 12-month-old mice, respectively. Each point represents the mean ± SEM, N = 6–8 for each point. (B) At ZT18 the STR significantly increased (−4.60 log log cd*s/m^2^ vs −3.60 log cd*s/m^2^n) in 12-month-old mice (t-tests, P<0.05). Figures C through E show representative traces of the ERG at the different ages (C = 3 months; D = 6 months; E = 12 months).

The scotopic ERG was recorded in mice of different ages (3, 6, and 12 months) and at two different time points (ZT6 and ZT18). As shown in Figures 1A through 1F, the amplitude of the a and b waves at ZT6 and ZT18 steadily decreases with age. The amplitude of the waves of younger mice (3 months) is significantly higher than that observed in 6-month-old mice (Two-way ANOVA, P<0.01 followed by Tukey tests, P<0.05). The amplitude of the a and b waves of 12-month-old mice was significantly lower than that measured in 6-month-old mice (Two-way ANOVA, P<0.01 followed by Tukey tests, P<0.05). No further decrease in the amplitude of a and b waves was observed in older mice (Two-way ANOVA, P>0.1, data not shown). The diurnal rhythm in the amplitude of the a and b waves was present in young mice (3 and 6 months, Two-way Anova, P<0.01; see Figure 1A, 1B, 1D and 1E) but not in older mice (Figures 1C and 1F; Two-way ANOVA, P>0.1). We then investigated the STR for the three different ages at ZT6 (Figure 2A) and at ZT18 (Figure 2B). As shown in Figure 2, no differences were observed at ZT6 between the three age groups (Two-way ANOVA, P>0.05); however, at ZT18, the STR for 3- and 6-month-old mice became detectable at the light intensity of −4.60 log cd*s/m^2^ (t-tests, P<0.05), while the light response of 12-month-old mice became detectable at −3.60 log cd*s/m^2^ (t-test, P<0.05).

### Effects of Aging on the Photopic ERG

**Figure 3:**
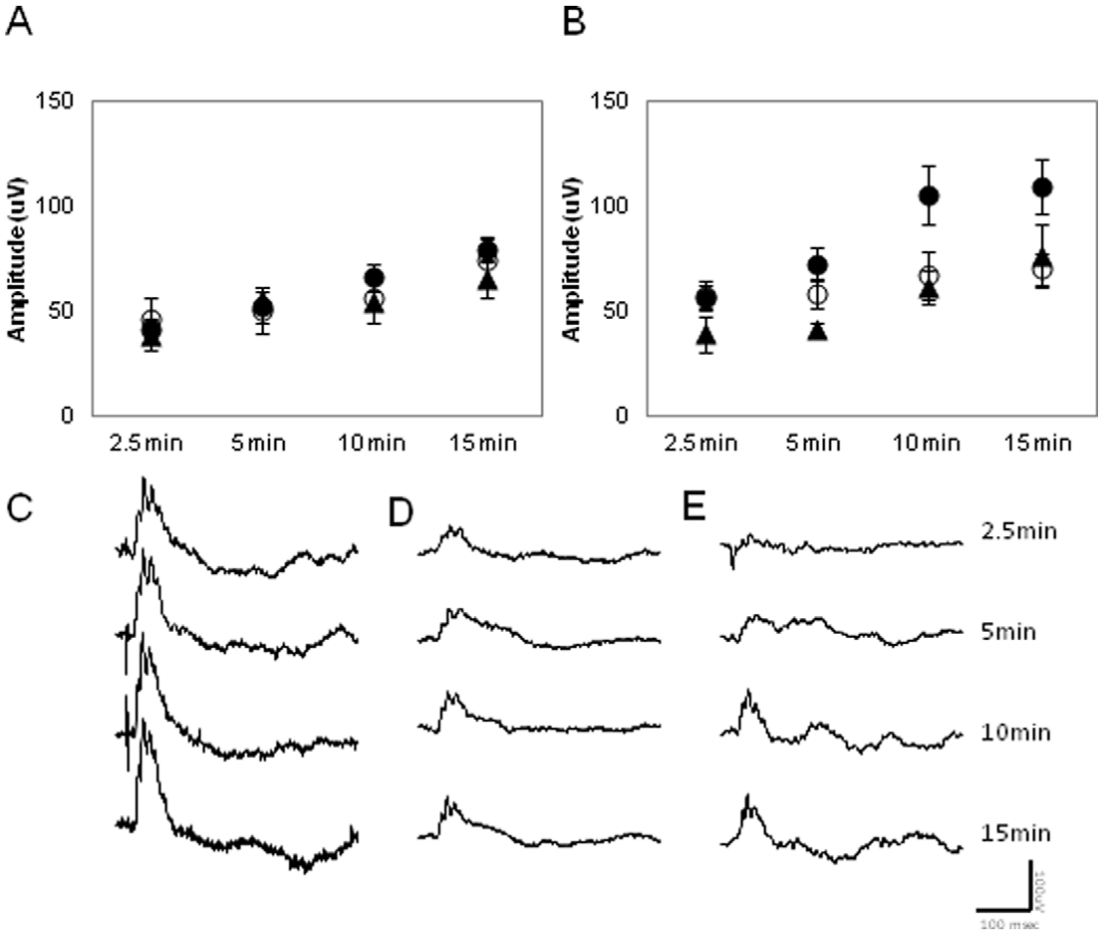
Effect of aging on the photopic ERG and its daily rhythm. (A) The amplitude of b wave of the photopic ERG at ZT6 was not affected by age at ZT6 (Two-way ANOVA, P>0.1). (B) It showed a significant decrease at ZT18 (Two-way ANOVA, P, 0.01, followed by Tukey tests, P<0.05) A significant difference in the amplitude of the b wave between ZT6 and ZT18 was only detected in 3-month-old mice (Two-way ANOVA, P>0.1 followed by Tukey tests, P<0.05). Closed circles, open circles, and closed triangle indicate 3-, 6- and 12-month-old mice, respectively. Each data point represents 4 to 8 animals, and the mean ± SEM, where N = 6–8 for each point. Figures C through E show representative wave forms of each age group at ZT18 (C = 3 months; D = 6 months and E = 12 months).

The effects of aging on the photopic ERG were investigated using a protocol similar to that used by Baba et al. [4]. The ERGs from C3H/f^+/+^ mice at different ages (3, 6 and 12 months) were recorded at ZT6 and ZT18 after 2.5, 5, 10, and 15 min of rod saturating background light exposure. Our data indicate that the amplitude of the b wave at ZT6 was not affected by age (Figure 3A, Two-way ANOVA, P>0.05). whereas the amplitude of the b wave at ZT 18 was significantly affected by age (Two-way ANOVA, P>0.01 followed by Tukey tests, P<0.05). The amplitude of the b wave of younger mice (3 months) at ZT18 was significantly higher than that observed in 6- and 12-month-old mice (Two-way ANOVA, P<0.01 followed by Tukey tests, P<0.05, Figure 3B).

### Effects of Aging on Retinal Organization

**Figure 4:**
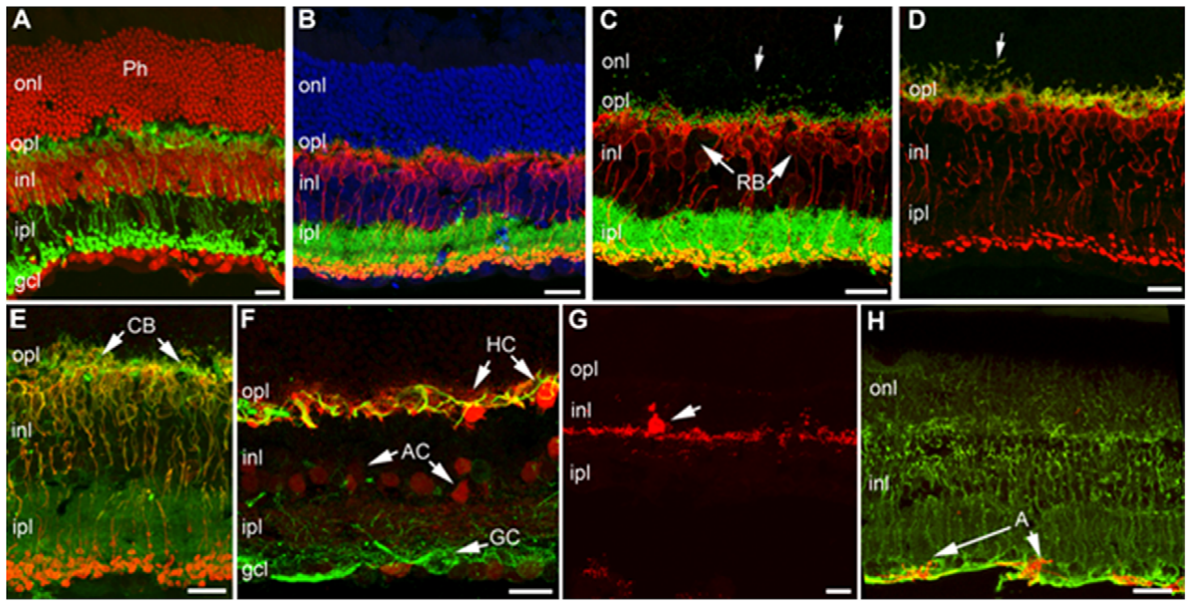
Retinal organization in C3H-f+/+ at 12 months of age. TOTO-3 staining of nuclear layers (red in A and blue in B) reveals an intact retinal layering in C3H-f^+/+^ control 12-month-old mice. (A) Rod bipolar cells labelled by protein kinase c (PKCα) antibodies (green) have normal morphology and layering. (B) PKCα antibodies show again RB (red) with normally distributed bassoon puncta (green) decorating their dendritic tips in the opl. (C) Rod bipolar cells (RB) (red) have dendritic tips associated to green puncta, representing kinesins II positive synaptic ribbons. Most puncta are appropriately confined in the opl, but some of them are displaced in the onl (arrows). (D) PKCα labelling of Rod bipolar cells (red) and PSD-95 (green). Most presynaptic endings of photoreceptors (labelled by PSD-95) are confined to the OPL, but a few are clearly seen in the onl (arrow). (E) Antibodies against Goα (green), specific for depolarizing bipolar cells, in combination with PKCα (red) allow visualization of rod bipolar cells (yellow-orange) and depolarizing cone bipolars (green, CB). Dendrites of both categories of cells are clearly visible (arrows). (F) Calbindin (red) in combination with neurofilament (green) antibodies show a normal pattern of staining of horizontal cell bodies (HC), their axonal endings (yellow profiles in the opl) and calbindin positive amacrine cells (AC). Neurofilament antibodies also stains ganglion cells (GC, arrow). (G) One tyrosine hydroxylase (TH) positive amacrine cell with the typical large size body (arrow) and main dendritic plexus in the outermost part of the ipl. (H) Antibodies against the enzyme glutamine synthase show the fine morphology of Müller glial cells (green). Anti-glial fibrillary acidic protein (GFAP) antibodies show astrocytes regularly placed at the innermost retinal margin (red, arrow). Scale bars are 20 μm. Ph: Photoreceptors; onl: outer nuclear; opl: outer plexiform; inl: inner nuclear; ipl: inner plexiform; gcl: ganglion cell layer.

We next investigated whether overall retinal organization and connectivity were affected by aging. No major alterations in retinal morphology were evident between retinas obtained from 3- and 12-month-old mice (Figure 4). Cells belonging to all retinal classes, including rods and cones, horizontal cells, rod and cone bipolar cells, various types of amacrine cells, such as Tyroxine Hydroxylase (TH) and calbindin positive amacrines, and ganglion cells, as well as Muller glia and astrocytes, were visualized with specific antibodies on vertical retinal sections and appeared normal in morphology, pattern of stratification, and overall architecture, conforming to the published literature [19]. Synaptic markers, namely bassoon, kinesin II, post-synaptic density protein 95 (PSD95), and synaptophysin, were normally distributed in the two plexiform layers of both genotypes. Local signs of focal rearrangements (i.e., formation of rosettes, a common finding in retinal reorganization) or signs of glial reactivity in Muller cells, microglia and astrocytes, which are frequently reported in a variety of pathological mutants [20], were undetected in these mice. The only evident feature at 1 year of age was the locally irregular profile of the outer plexiform layer, which was particularly visible when markers such as PSD95 or kinesin 2 and bassoon (labeling photoreceptor synaptic endings and ribbon proteins, respectively) were used. These antibodies showed immunopositive elements in the outer nuclear layer (i.e. panels C, D and E of Figure 4), indicating a mislocation of the photoreceptor endings and their synaptic machinery toward the outer retina. We explain this finding as the beginning of the known process of retraction of photoreceptor endings, paralleled by sprouting of bipolar cell dendrites common in the retina of very old mice [21] but starting at one year of age [22].

### Effects of Administration of Exogenous Melatonin on the Scotopic ERGs

**Figure 5:**
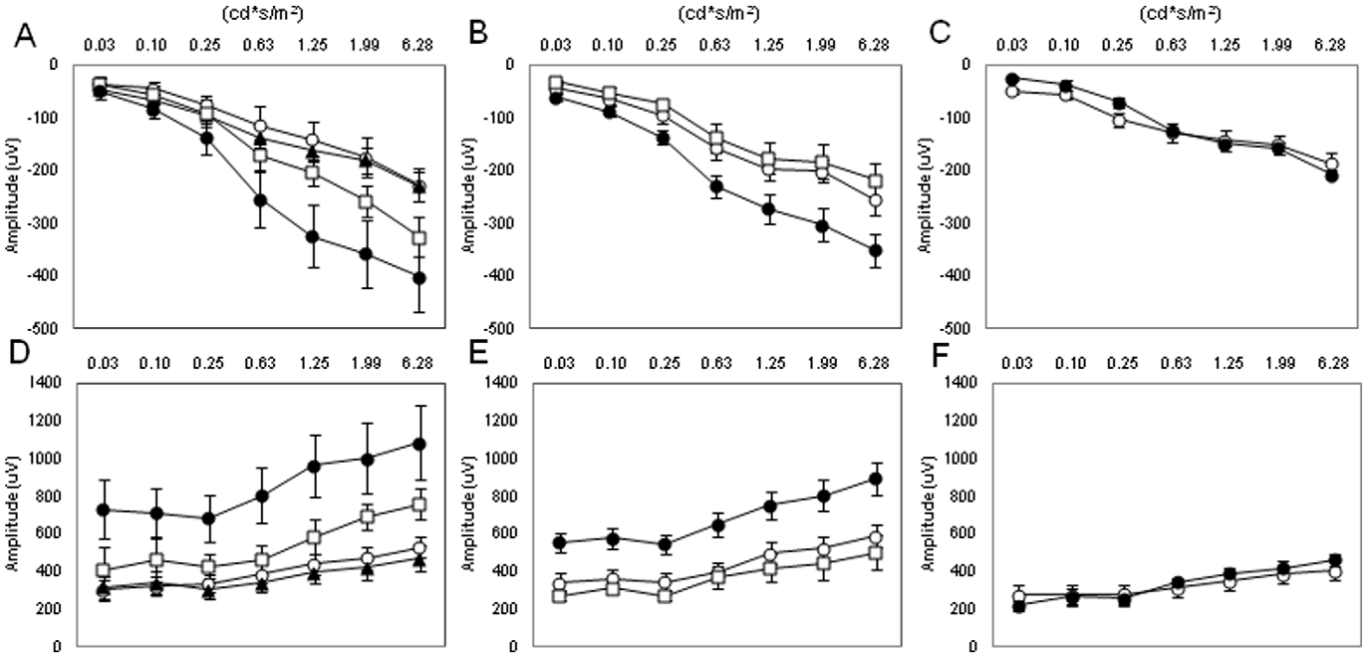
Effect of administration of exogenous melatonin on the scotopic ERG at different ages. Melatonin injection (1 mg/Kg) increased the amplitude of the a (A, B) and b wave (D, E) of the scotopic ERG in 3- and 6-month-old mice (Two-way ANOVA P<0.01, followed by Tukey tests, P<0.05) with respect to the values obtained in control mice. The same dose did not increase the amplitude of the a and b wave in 12 months old mice (Two-way ANOVA, P>0.01, C, F). Melatonin injection (0.1 mg/Kg) increased the amplitude of the a and b waves of the scotopic ERG in 3-month-old mice (Two-way ANOVA, P<0.01, followed by Tukey tests, P<0.05 A, D), but not in 6 month-old mice (Two-way ANOVA, P>0.05; B, E, C, F). White circles indicate control groups; black triangles indicate melatonin 0.01 mg/kg; white squares indicate melatonin 0.1 mg/kg; and black circles indicate melatonin 1 mg/kg. Each value represents the mean ± SEM, N = 5–6 for each point.

We previously showed that administration of exogenous melatonin (1 mg/kg i.p.) induces a significant increase in the amplitudes of dark-adapted ERG in young mice; specifically, injections of 1 mg/kg of melatonin at ZT 6 can increase the amplitude of a and b waves in 3-month-old mice (P<0.01, Two-way ANOVA, Figure 5A, D) [4]. In this study, we tested whether injections of exogenous melatonin could restore the ERG amplitude to the levels of young mice by increasing the concentration of melatonin (0.01, 0.1 and 1 mg/Kg). In 3-month-old mice, injection of 1 and 0.1 mg/Kg of melatonin at the dose increased the amplitude of the a and bwaves of the scotopic ERG (Two-way ANOVA, P>0.05, followed by Tukey tests, P<0.05, Figures 5A and 5D). No effect was observed with administration of 0.01 mg/Kg (Two-way ANOVA, P<0.1). The dose of 0.1 mg/kg did not have an effect on a and b wave amplitude in 6-month-old mice (Two-way ANOVA, P>0.05, Figures 5B and 5E). Injecting 1mg/kg of melatonin increased the amplitude of the a wave (Two-way ANOVA p<0.05 followed by Tukey tests, P<0.05, melatonin vs. control, Figures 5B and 5E) and of the b wave (Two-way ANOVA P<0.01 followed by Tukey tests, P<0.05, melatonin vs. control, Figures 5B and 5E) in 6-month-old mice. However, it did not increase the amplitude of the a and b waves in 12-month-old mice (Two-way ANOVA, P>0.1 melatonin vs. control, Figures C, F). Although melatonin administered to 6-month-old mice successfully recovered the amplitude of the a -wave, it did not fully rescue the amplitude of the b wave (3 months vs. 6 months; Two-way ANOVA p<0.05, followed by Tukey tests, P<0.05). The same melatonin treatment increased both a and b wave amplitudes in 3-month-old mice (a wave p<0.05, b wave, Two-way ANOVA, p<0.01control vs. 0.1 mg/kg).

## Discussion

The role played by melatonin and melatonin receptors in the mammalian retina is not well defined. The lack of data is due to the fact that the vast majority of mouse strains are genetically deficient in synthesizing melatonin in the pineal gland and retina [20]–[21], so very few studies have compared retinal physiology in melatonin-proficient and melatonin-deficient mice. This situation is further complicated by the fact that C57/BL6 and other strains may produce a small amount of melatonin; therefore, we cannot completely exclude that this small amount may somehow influence the mouse physiology [23]. In the present study, we investigated the effect of aging on retinal function and morphology in a melatonin proficient mouse strain (C3H-f^+/+^). Our data indicate that the following: the amplitude of the a and b waves of dark-adapted ERG decrease with age; the daily rhythm in the ERGs is lost by the age of 12 months; the STR at ZT18 is significantly affected by age; the amplitude of the photopic ERG and its daily rhythm are also affected by age; the changes observed in the ERGs are not paralleled by gross alterations in retinal general morphology; and administration of exogenous melatonin affects the ERGs in 12-month-old mice.

Age-related declines in retinal function have been reported for mice [13]–[14] and for humans [24]. Our data are in agreement with previous reports and expand these results by showing that the daily rhythm in the scotopic and photopic ERGs and the STR are both affected by aging (Figures 1, 2, 3). Interestingly, no further decrease in the amplitude of the a and b waves was observed after 12 months of age for both scotopic and photopic conditions. The observation that the STR is only affected by aging at ZT18 is not surprising, since mice are nocturnal and therefore the visual system is optimized to perform at night when the mice are active.

Previous studies have shown that photoreceptor loss is only observed in mice older than 12 months [4], [13], [14] with no significant changes observed before this age [4], [13]. Our immunocitochemistry analysis (Figures 4) indicates that many of the retinal biomarkers are not affected by aging in C3H-f^+/+^ mice. In our retina samples of 12 months age, the only effect seen in the retina (i.e., the retraction of the photoreceptor and sprouting of the bipolar cells dendrites) is congruent with the aging signature already reported in literature [21], [22]. This result suggests the future search and quantification of markers, which might help explain our ERG finding, will require a detailed analysis on other components of the signaling pathways, such as the amount of rhodopsin or of other phototransduction proteins, the distribution of mGluR6, or the number of rod and on-cone bipolar cells. Our data also indicate that the decrease in retinal functioning (ERG) and sensitivity (STR) is important for the early detection of photoreceptor dysfunctions, preceding any obvious morphological change in retinal organization.

Several investigations have shown that blood melatonin levels or biosynthesis decline with age [18]. It has been also reported that a similar scenario may be present in the retina since the level of Arylalkylamine N-acetyltransferase, a key enzyme for melatonin synthesis, transcription or protein expression, decreases with age [25], [26]. Our previous work showed that in melatonin-proficient mice, the daily rhythms observed in ERG parameters are controlled by melatonin [4]; therefore, we hypothesized that the observed decline in the amplitudes of the ERG and the loss of the daily rhythm may be due to the decline of melatonin synthesis in older mice. To test this, we administered exogenous melatonin to mice of different ages. Surprisingly, we observed a dose- and age- dependent increase in the responsiveness of the ERG to administration of exogenous melatonin (Figure 5), which was not rescued by exogenous melatonin. Such a result may suggest that the reduction in the melatonin receptors is responsible for the lack of responsiveness to the administration of exogenous melatonin. Interestingly, a previous study has reported a loss of responsiveness to administration of exogenous melatonin in the suprachiasmatic nucleus (SCN) of aged mice [27]; in addition, melatonin receptor expression decreases during aging in human SCN and in patients affected by Alzheimer's [28] and Parkinson's diseases [29]. Such results may suggest that the lack of responsiveness to the administration of exogenous melatonin is probably due a reduction in the levels of melatonin receptors present in the photoreceptors. Further studies will be required to address this important issue.

Melatonin and its analogues are currently used by millions of people around the world to prevent aging, to improve sleep performance, to ameliorate jet-lag symptoms and to treat depression [30]. Our new study indicates that responsiveness to exogenous melatonin is affected by aging; therefore, in some instances, melatonin treatment may not be effective due to lack or reduced sensitivity of its receptors.

## Materials and Methods

### Animals

C3H/f^+/+^ mice used in this study were bred and maintained at Morehouse School of Medicine in a 12-h light/12-h dark cycle, with lights on from zeitgeber time (ZT) 0 to ZT 12 with food and water *ad libitum*. All experiments conformed to the NIH Guide on the Care and Use of Laboratory Animals, and were approved by the Institutional Animal Care and Use Committees of Morehouse School of Medicine.

### Electroretinography

Mice were anesthetized with ketamine (80 mg/kg) and xylazine (16 mg/kg). The pupils were dilated with 1% atropine and 2.5% phenylephrine (Sigma, St. Louis, MO, USA), and mice were placed on a regulated heating pad set at 37°C with feedback from the rectal temperature probe. The eye was lubricated with saline solution, and a contact lens type electrode (LKC Technologies model: N1530NNC) was topically applied on the cornea. A needle reference was inserted in other side of cheek, and the ground needle was inserted into the base of tail. All preparation of ERG recordings was conducted under red dim light (<3 lux, 15 W Kodak safe lamp filter 1A, Eastman Kodak, Rochester, NY, USA).

All electrodes were connected to a Universal DC Amplifier (LKC Technologies model UBA-4200) and bands were filtered from 0.3 to 500 Hz. Data were recorded and analyzed by EM for Windows (ver. 8.2.1, LKC Technologies). Core body temperature was maintained in 37°C by a feedback temperature control system (FHC inc., Bowdoin, ME) during whole ERG recording. In the dark-adapted ERG protocol, seven series of flash intensity between from 0.03 to 6.28 cd*s/m^2^ were presented to the mouse eye. Flashes were generated by 530-nm green LEDs in a Ganzfeld illuminator (LKC Technologies), and intervals of flashes increased from 0.612 to 30 s as intensity of the flashes increased. Responses of 3–10 flashes were averaged to generate a waveform for each step of light intensity, and a-wave and b-wave of ERG measurement were analyzed from the trace of wave forms.

To measure the photopic ERG mice were placed in a Ganzfeld illuminator and cone-associated activity was isolated by saturating rods with 63 cd*s/m^2^ of white background light. The four series of consecutive 10 white flashes (79.06 cd*s/m^2^) were introduced at 2.5 min, 5 min, 10 min, and 15 min during the background light exposure. Background light was left on for 15 min while photopic ERGs records were measured [4]. The traces of the ERG were averaged and stored on a computer for later analysis. The amplitude of the b-wave was measured from the trough of the a-wave to the peak of the b-wave or, if no a-wave was present, from the baseline to the b-wave peak. The spectral composition and irradiance of the light was monitored by a radio-spectrophotometer (USB 2000, Ocean Optics, Dunedin, FL). The STR was determined using the same protocol described in Baba et al., [4].

### Immunocytochemistry

Details about the antibodies and immunocytochemistry procedures used to study retinal organization are described in detail in published work [4], [31].

### Administration of Exogenous Melatonin

Melatonin (Sigma, St Louis, MO) was dissolved in ethanol and then diluted with sterilized PBS in volume of 10 mL/kg. The solution was administered to mice by intraperitoneal (i.p.) injection at 1 mg/kg if not otherwise indicated. The same volume of vehicle alone was injected to the control group animals Melatonin or vehicle was injected 1h before ERG recordings just before mice were placed in the dark isolated chamber. All melatonin injections were given at ZT 5 when endogenous melatonin level is undetectable.

### Statistical Analysis

Comparison among the values obtained with the mice at different ages and light intensity were performed using a Two-vay Analysis of Variance (variable 1: age and variable 2: luminance) when the analysis of variance indicated a significant effects of age and light intensity (P<0.05) and a non significant interaction between age and luminance (P>0.05) we performed a multiple comparison test (Tukey tests, P<0.05). The STR was determined using a t-test as described in [4]. All the statistical tests were performed using a statistical package (Sigma-Stat, 3.5).

## References

[1] Tosini G, Davidson AJ, Fukuhara C, Kasamatsu M, Castanon-Cervantes O (2007). Localization of a circadian clock in mammalian photoreceptors. FASEB J..

[2] Rufiange M, Dumont M, Lachappelle P (2002). Correlating retinal function with melatonin secretion in subject with an early or late circadian phase. Invest Ophthalmol Vis Sci..

[3] Peters JL, Cassone VM (2005). Melatonin regulates circadian electroretinogram rhythms in a dose- and time-dependent fashion. J Pineal Res..

[4] Baba K, Pozdeyev N, Mazzoni F, Contreras-Alcantara S, Liu C (2009). Melatonin modulates visual function and cell viability in the mouse retina via the MT1 melatonin receptor.. Proc Natl Acad Sci U S A.

[5] Gagne AM, Danilenko KV, Rosolen SG, Herbert M (2009). Impact of oral melatonin on the electroretinogram.. J Circadian Rhythms.

[6] Sugawara T, Sieving PA, luvone PM, Bush RA (1998). The melatonin antagonist luzindole protects retinal photoreceptors from light damage in the rat. Invest Ophthalmol Vis Sci..

[7] Liang FQ, Aleman TS, Yang Z, Cideciyan AV, Jacobson SG (2001). Melatonin delays photoreceptor degeneration in the rds/rds mouse.. Neuroreport.

[8] Yi C, Pan X, Yan H, Guo M, Pierpaoli W (2005). Effects of melatonin in age-related macular degeneration. Ann N Y Acad Sci..

[9] Rosen R, Hu DN, Perez V, Tai K, Yu GP (2009). Urinary 6-sulfatoxymelatonin level in age-related macular degeneration patients. Mol Vis..

[10] Fujieda H, Hamadanizadeh SA, Wankiewicz E, Pang SF, Brown GM (1999). Expression of mt1 melatonin receptor in rat retina: evidence for multiple cell targets for melatonin.. Neuroscience.

[11] Sengupta A, Baba K, Mazzoni F, Pozdeyev NV, Strettoi E (2011). Localization of melatonin receptor 1 in mouse retina and its role in the circadian regulation of the electroretinogram and dopamine levels. PLoS One..

[12] Meyer P, Pache M, Loeffler KU, Brydon L, Jockers R (2002). Melatonin MT-1-receptor immunoreactivity in the human eye.. Br J Ophthalmol.

[13] Li C, Cheng M, Yang H, Peachey NS, Naash MI (2001). Age-related changes in the mouse outer retina. Optom Vis Sci..

[14] Gresh J, Goletz PW, Crouch RK, Rohrer B (2003). Structure-function analysis of rods and cones in juvenile, adult, and aged C57bl/6 and Balb/c mice. Vis Neurosci..

[15] Goto M, Oshima I, Tomita T, Ebihara S (1989). Melatonin content of the pineal gland in different mouse strains. J. Pineal Res..

[16] Tosini G, Menaker M (1998). The clock in the mouse retina: melatonin synthesis and photoreceptor degeneration. Brain Res..

[17] Roseboom PH, Namboodiri MA, Zimonjic DB, Popescu NC, Rodriguez IR (1998). Natural melatonin ‘knockdown’ in C57BL/6J mice: rare mechanism truncates serotonin N-acetyltransferase. Brain Res Mol Brain Res..

[18] Bubenik GA, Konturek SJ (2011). Melatonin and aging: prospects for human treatment. J Physiol Pharmacol..

[19] Jeon CJ, Strettoi E, Masland RH (1998). The major cell populations of the mouse retina. J Neurosci..

[20] Damiani D, Alexander JJ, O'Rourke JR, McManus M, Jadhav AP (2008). Dicer inactivation leads to progressive functional and structural degeneration of the mouse retina. J Neurosci..

[21] Liets LC, Eliasieh K, van der List DA, Chalupa LM (2006). Dendrites of rod bipolar cells sprout in normal aging retina. Proc Natl Acad Sci U S A..

[22] Terzibasi E, Calamusa M, Novelli E, Domenici L, Strettoi E (2009). Age-dependent remodelling of retinal circuitry.. Neurobiol Aging.

[23] Vivien-Roels B, Malan A, Rettori MC, Delagrange P, Jeanniot JP (1998). Daily variations in pineal melatonin concentrations in inbred and outbred mice. J Biol Rhythms..

[24] Birch DG, Anderson JL (1992). Standardized full-field electroretinography. Normal values and their variation with age. Arch Ophthalmol..

[25] Pulido O, Clifford J (1986). Age-associated changes in the circadian rhythm of retinal N-acetylserotonin and melatonin in rats with pigmented eyes. Exp Gerontol..

[26] Tosini G, Chaurasia SS, Iuvone PM (2006). Regulation of AANAT in the retina.. Chronobiology International.

[27] Von Gall C, Weaver DR (2008). Loss of responsiveness to melatonin in the aging mouse suprachiasmatic nucleus. Neurobiol Aging..

[28] Wu YH, Zhou JN, Van Heerikhuize J, Jockers R, Swaab DF (2007). Decreased MT1 melatonin receptor expression in the suprachiasmatic nucleus in aging and Alzheimer's disease.. Neurobiol Aging.

[29] Adi N, Mash DC, Ali Y, Singer C, Shehadeh L (2010). Melatonin MT1 and MT2 receptor expression in Parkinson's disease. Med Sci Monit..

[30] Sánchez-Barceló EJ, Mediavilla MD, Tan DX, Reiter RJ (2010). Clinical uses of melatonin: evaluation of human trials. Curr Med Chem..

[31] Gargini C, Terzibasi E, Mazzoni F, Strettoi E (2007). Retinal organization in the retinal degeneration 10 (rd10) mutant mouse: a morphological and ERG study.. J Comp Neurol.

